# Content validity of the Scoliosis Research Society questionnaire (SRS-22r): A qualitative concept elicitation study

**DOI:** 10.1371/journal.pone.0285538

**Published:** 2023-05-05

**Authors:** Samia Alamrani, Adrian Gardner, Deborah Falla, Emily Russell, Alison B. Rushton, Nicola R. Heneghan

**Affiliations:** 1 Centre of Precision Rehabilitation for Spinal Pain (CPR Spine), School of Sport, Exercise and Rehabilitation Sciences, College of Life and Environmental Sciences, University of Birmingham, Birmingham, United Kingdom; 2 Physical Therapy Department, College of Applied Medical Science, University of Tabuk, Tabuk, Saudi Arabia; 3 Spine Unit, The Royal Orthopaedic Hospital, Northfield, Birmingham, United Kingdom; 4 Oxford University Hospitals NHS Foundation Trust, Oxford, United Kingdom; 5 School of Physical Therapy, Western University, London, Ontario, Canada; Nnamdi Azikiwe University, NIGERIA

## Abstract

**Introduction:**

Scoliosis Research Society-22 revised (SRS-22r) is the common questionnaire used to evaluate health related quality of life (HRQOL) for young people with adolescent idiopathic scoliosis (AIS). The aim of this study is to evaluate its content validity for this population.

**Methods:**

In-depth semi-structured interviews were conducted with a purposive sample of young people with AIS (Cobb angle ≥25˚, aged 10–18 years). Concept elicitation was used to evaluate the influence of AIS on participants’ HRQOL. Participant information sheets and consent/assent forms were age relevant. Topic guide was informed by the SRS-22r and existing evidence. Interviews were audio and video recorded, transcribed verbatim, coded, and analysed using thematic analysis. Derived themes/codes were compared with SRS-22r contents (domains/items).

**Results:**

Eleven participants (mean age 14.9 years [SD = 1.8]; 8 female) were recruited. The mean curve size was 47.5° [SD = 18°] and participants had been managed via different approaches. Four main themes emerged with associated subthemes: 1) Physical effects related to physical symptoms (back hurt, stiffness) and body asymmetry (uneven shoulders), 2) Activity-related effects showed impact on mobility (sitting for long periods), self-care (dressing), and school activities (focus during lessons), 3) Psychological effects revealed emotional (feel worried), mental (sleep quality), and body image effects (hide back from others), 4) Social effects (participation in school and leisure activities), and school, friends and mental health support. A weak association was found between items of the SRS-22r and the identified codes.

**Conclusion:**

The SRS-22r does not adequately capture important concepts that relate to HRQOL of adolescents with AIS. These findings support revision of the SRS-22r, or the development of a new patient reported outcome measure to evaluate HRQOL of adolescents with AIS.

## Introduction

Scoliosis Research Society (SRS) questionnaire is the common patient reported outcome measure (PROM) for young people with adolescent idiopathic scoliosis (AIS) [1, 2]. It was developed by Haher et al. in 1999 by incorporating questions from previous questionnaires used to evaluate surgical treatment of adults with scoliosis [3]. The developed questionnaire (SRS-24) was refined and updated to enhance its internal consistency, and two items were removed producing the SRS-22r [4]. The SRS-22r has been included in the Core Outcome Set (COS) for adolescents and young adults with spinal deformity to evaluate self-image, physical functioning and pain [5].

As directed by the initiatives of the Core Outcome Measures in Effectiveness Trials (COMET) consortium, if the SRS-22r has been recommended as a PROM to be selected in a COS, it should exhibit at least high-quality evidence of good content validity [6]. Content validity is the first, and most important measurement property to consider when selecting any PROM [6]. Adequate content validity indicates that the contents of a PROM are consistent with the perspective, words, and experiences of the population of interest [7, 8]. The use of a PROM that does not appropriately reflect the population being measured might undermine the validity of study findings. On the other hand, using a PROM having content validity for the intended purpose and other essential psychometric qualities can lead to advances in theory and practise [9]. Qualitative research methods such as the use of concept elicitation methodology may usefully assess content validity by interviewing the population of interest to understand their perspectives regarding the impact of the disease on their own health condition [10–12].

The psychometric properties of the SRS-22r have been extensively studied [4, 13–15], with the SRS-22r translated and culturally adapted into more than ten languages [1]. However, participants in these studies were older than 18 years, (range 19–34 years), and therefore they may not be representative of an AIS population. Furthermore, the mental health domain of the SRS-22r includes questions from the SF-36 survey, which is a generic questionnaire designed for adults [15]. The SRS-22r is reported to have ceiling effects (20–44%) [4], which limit the reported content validity and reliability [6]. The Scoliosis Quality of Life Index (SQOLI) is another PROM which was developed to address the limitations of the SRS-22r and improve relevance to AIS [16]. However, the SQOLI is not widely utilised, and the SRS-22r remains the commonly used PROM for health-related quality of life (HRQOL) evaluation in people with AIS. Because adolescence is a distinct demographic age group with discrete emotional and social characteristics, it is essential that their PROM represents their developmental stage [17].

A recent scoping review exploring the experiences of AIS and families about the diagnosis and treatment of scoliosis revealed a need for more qualitative research for this population [18]. The majority of studies evaluated decision for treatment [19] and focused on the experience of surgery [20], mainly its effects on the psychological aspects for the patient and parents [21, 22].

To the best of our knowledge, no previous qualitative study has interviewed individuals with AIS to elicit concepts about their scoliosis and their impact on HRQOL. Evidence for content validity of the SRS-22r is therefore lacking. The aim of this study was to determine whether the content of the SRS-22r questionnaire is relevant and important to participants with AIS, by exploring the impact of scoliosis and its associated treatment on HRQOL.

## Methods

### Ethics approval

Ethical approval to conduct the study was sought from the Health Research Authority and Health and Care Research Wales approval (REC reference: 21/WM/0076).

### Design

This qualitative study design is reported in line with the COnsolidated criteria for Reporting qualitative study [23], and has been described in a published study protocol [1].

### Theoretical framework

Phenomenology was used as theoretical framework and adapted grounded theory as the methodology for collecting data and analysis [11, 24, 25]. Phenomenology is a philosophical approach focusing on the experience the individual experiencing the phenomenon [26]. It was used to explore in depth adolescents’ lived experiences with idiopathic scoliosis and the related treatments. Adapted ground theory is an approach in which previous knowledge grounded in existing literature and expert opinion is used to develop domains and probes in the topic guide [7]. It provides a framework for data generation and coding which guide analysis process [27]. The existing information about the SRS-22r, was used to inform study design, the topic guide, and to identify themes and concepts (domains and items) and to interpret the results [24].

### Participant selection

A heterogenous purposive sampling approach was used to recruit individuals with AIS aiming for n = 10–15, which is deemed sufficient to reach concept saturation [28]. This approach enabled a wide range of experience and opinions to be explored and ensured diversity in the sample characteristics [29]. Participants with a Cobb angle >25°, aged 10–18 years, and who had access to a video/audio call platform, were considered eligible. Individuals with other forms of scoliosis, and those who were unable to speak English fluently were excluded.

### Data collection and setting

Participants were recruited from a tertiary scoliosis centre in the United Kingdom. Due to COVID-19 restrictions semi-structured interviews were conducted virtually (using Zoom/ Microsoft Teams platforms). Written consents to participate in the study were collected from participants and parents by the research nurse, during their visit to the clinic. The lead researcher (SA) contacted the participant/parent, and the interview time was chosen based on participant/parent preferences. Data collection took place between January and April 2022.

### Topic guide

**Fig 1:**
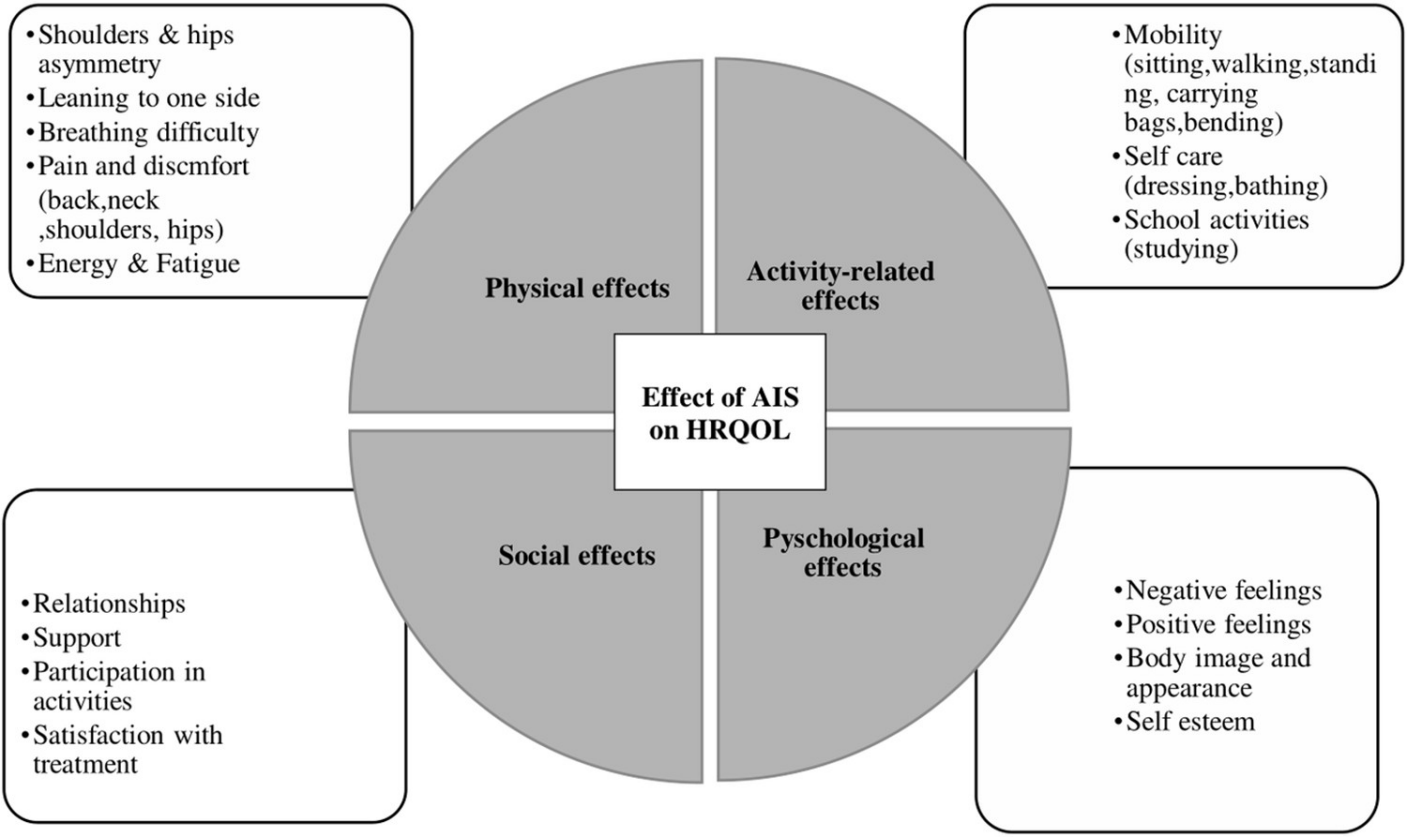
Hypothesised conceptual framework using HRQOL dimensions.

The HRQOL concept is a “complex, multidimensional concept, including social, emotional and physical functioning or well-being, related to the patient’s health state” [30]. To ensure that the concept of HRQOL was elicited from participants during the interview, a topic guide was developed using a hypothesised conceptual framework (Fig 1), as well as the recommended guidelines on the development of PROM for measuring HRQOL [31, 32]. The topic guide was age appropriate and consisted of open-ended questions that explored concepts of interest (i.e., HRQOL) as well as other concepts from the SRS-22r such as satisfaction. Ended by exploring other areas that are important to participants with AIS, such as participation in sport and other physical activities (S1 Appendix).

### Protocol amendments

An amendment was made to the topic guide after piloting with a participant with AIS. The revision was to improve clarity of some questions, as they may lead to confusion. Furthermore, details about confidentiality were explained and simplified.

### Interview procedure

At the beginning of the interview, the interview format was explained, and any questions were answered. The confidentiality, voluntary participation and withdrawal process was also explained. Demographic and clinical data were collected electronically before the interviews took place. Participants were encouraged to talk for as long as was needed during the interview, which lasted a mean time of 49 minutes (SD = 9.7). Participants were unknown to the interviewer (SA) prior to the interview in all cases. The interviewer and participants were alone during interview in all but three instances, where the interview was observed by a parent. Interviews were audio and video recorded, and field notes were collected following the interview. Audio recordings were transcribed verbatim by an official transcription service. Interview transcripts were sent to participants to check accuracy with an opportunity for participants to add any further details if required. No revisions or amendments were received.

### Research team and reflexivity

An experienced musculoskeletal physiotherapist researcher (SA) conducted the interviews, with support from specialist co-authors in spinal research (surgeon, physiotherapists), and qualitative research. No specific relationship was established prior to the commencement of the interviews and participants were informed about the professional background of the interviewer and that the study is part of a PhD thesis.

### Patient and public involvement (PPI)

This study was conceived as a direct consequence of gaps identified in a previous systematic review of physical functioning outcome measures amongst participants with AIS [1]. The PPI representative was part of the study management group, and her feedback had been sought on the study protocol, the topic guide as well as versions of the participant information sheet and consent forms. Furthermore, the PPI representative was involved in data analysis and interpretation of study findings through provision of a plain English summary.

### Data storage and management

All study investigators complied with requirements of the Data Protection Act 2018 with regards to the collection, storage, processing and disclosure of personal information and upheld the Act’s core principles. Personal data were coded and depersonalised and replaced with a participant identification number. and stored electronically on a password-protected computer at the University of Birmingham. Secure maintenance of the data ensured that the linking code were kept securely in a separate location using encrypted digital files within password- protected folders and storage media. Only the Chief Investigator and the study Research Fellow had access to the data as necessary for quality control, audit and analysis. Data will be stored for 10 years in line with the University of Birmingham’s Research Governance procedures only accessible for the research team.

### Data analysis

Data were analysed according to the recommended guidelines for the evaluation of the content validity of a PROM [7, 8, 24], with guidance from thematic analysis [33]. A coding framework was developed using the topic guide and the hypothesised conceptual framework [24]. Coding was performed by SA with guidance from the co-authors. A saturation table (S2 Appendix) was created to ensure that concept saturation was achieved, at the point where two consecutive interviews failed to elicit any new themes [28]. To ensure that the results were representative of the concept of interest among different participant characteristics or subgroups [8]. Coding was stratified according to participant characteristics (i.e., curve severity and management approach). Deductive analysis was used to organise the data into themes, which were predetermined by the topic guide or emerged from the data related to the concept [7]. Themes that emerged were mapped to the SRS-22r contents (domain and items) [8]. The words and phrases in the derived themes and subthemes were compared to words used in the SRS-22r questionnaire [7, 8]. If a poor agreement was present, indicating inadequate coverage of the concept of interest, an adaption to the PROM, or the development of a new PROM, should be made to support its use [8].

## Findings

### Participants

Demographic and clinical characteristics of participants are presented in Table 1. From 19 recruited and consented participants, 11 took part in the study. Four participants could not be contacted because they did not respond to the invitation from the researcher. Two withdrew due to school commitments and one withdrew because of parental withdrawal of consent. One participant withdrew once the interview had commenced as they denied speaking and participating because of feeling shyness. Therefore, their demographic data were discarded. The sample was representative of the population of adolescents with AIS, with the majority being female (n = 8), aged between 10–18 years old.

**Table 1:** Participants demographic and clinical characteristics.

ID	Age (years)	Gender	Curve severity	Location of curve	Lenke type	Management	Duration Following surgery	Number of X-rays	Pain intensity (0–10)	Duration of pain	Physical activity level per day	SRS-22r score Mean (SD)
1	15	Female	51°	Thoracic	1	Observation	NA	2	5	> 6 mos.	< 60 min.	3.2 (.89)
2	17	Female	32°*	Thoracic	3	Post-surgery	20 mos.	12	5	> 6 mos.	> 60 min.	3.7 (.72)
3	12	Female	31°	Thoracolumbar junction	5	Brace	NA	5	3	> 6 mos.	> 60 min.	4.4 (.46)
4	12	Female	25°	Thoracolumbar junction	5	Observation	NA	1	0	-	> 60 min.	4.6 (.81)
5	16	Female	40°*	Thoracic	1	Post-surgery	10 mos.	8	5	> 6 mos.	60 min.	4 (.76)
6	17	Male	57°	Thoracic	1	Pre-surgery	NA	6	4	> 6 mos.	< 60 min.	3.3 (.71)
7	16	Female	70°	Thoracic	1	Pre-surgery	NA	2	8	> 6 mos.	< 60 min.	3.7 (.52)
8	16	Female	70°	Thoracolumbar junction	1	Post-surgery	3 mos.	11	4	> 6 mos.	< 60 min.	3.4 (.68)
9	14	Male	40°	Thoracic	1	Brace	NA	2	3	3–6 mos.	< 60 min.	4.1 (.68)
10	13	Female	86°	Thoracic	1	Pre-surgery	NA	3	7	> 6 mos.	> 60 min.	2.8 (.66)
11	16	Male	40°	Thoracic	1	Pre-surgery	NA	2	7	> 6 mos.	60 min.	3 (.52)

* ^Indicates curve severity post-surgery^

**Fig 2:**
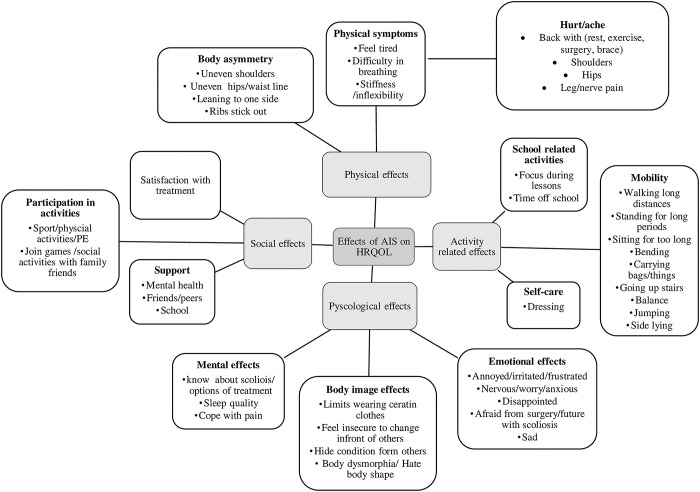
Updated conceptual framework.

A slight modification was made to the hypothesised conceptual framework (Fig 1), when compared to the updated conceptual framework (Fig 2) that was developed from the data. The identified themes were: (1) physical effects, (2) activity-related effects), (3) psychological effects, and (4) social effects. Some of the hypothesised subthemes were discarded as no data were collected to support their inclusion. Each theme and subthemes with appropriate code (participants quotes) are presented in (S3 Appendix).

### Physical effects

This theme was consistent with hypothesised subthemes across the two conceptual frameworks. In addition to various new subthemes were identified.

#### Physical symptoms

Back hurt was the main physical symptom reported by study participants. Those with severe curves expressed high pain intensity compared to participants with milder curves. Pain was associated with exercise performance such as stretching exercises as a form of sport (e.g., yoga), or in a form of treatment (e.g., physiotherapy). Participants noted that exercise was the leading cause of pain and the need for subsequent analgesia, which affected their participation in sport or physical education in school.

*“Sometimes it hurts my back when I do some exercises*.., *when I play football*, *it hurts my back”* (P11-pre-surgery)

Management of AIS (e.g., surgery) contributed also to the feel of pain. It affected school attendance, performance and the need for analgesia for several months following surgery. Bracing also can contribute to the experience of pain, and it was described as uncomfortable, and causes bruises.

*“Whereas now if I do get pain*, *it’s because of the surgery” (*P8-post surgery)

Participants also reported shoulder and hip pain, which was associated with body asymmetry. Shoulder and hip asymmetry were thought to affect being able to find a comfortable sleeping position and having a good quality of sleep.

*“I think just my shoulders and my hips sometimes because it’s kind of like disjointed everything*. *They ache most of the time and no matter how many medications I can take for it*, *it will always be there because it just reoccurs”* (P1-observation)

Nerve or leg pain was also reported by those with severe curves and waiting surgery, contributed to the feeling of weak legs and the increased need of taking pain medications.

*“When I get nerve pain*, *I have to take three [painkillers] to kill it” (*P10-Pre-surgery).

Participant reported feeling more tired when comparing themselves to their peers at school, and they felt that they needed to take more breaks in the day. Difficulty in breathing was also reported especially with running, and among those with severe curves.

*“I definitely feel more tired than other people after I do a lot of things and I have to take more breaks than most of my friends when they do the same things”* (P1-Observation).

Our study participants have not reported any limitation in range of movements; however, the terms “stiffened”, and ‘inflexibility’ were used by some.

*“I’m less flexible; I’ve never really been that flexible*. *I used to be really; I could do anything*. *But now it feels kind of stiffened and I’ve definitely lost quite a lot of flexibility”* (P1-observation)

#### Body asymmetry

Participants did not see or report a back hump that reflects the asymmetry of the posterior torso and is a physical sign of scoliosis, whilst a parent had noticed it. They noted different shoulder and hip levels, and with severe curves they noticed the ribs being more prominent. They also reported feelings of leaning over to one side.

*“My hips are about five centimetres apart so like one’s there*, *one’s there…”* (P10-presurgery)

### Activity-related effects

Three main themes were identified related to the effects of scoliosis on activity (mobility, self-care, school related activities). Compared with the hypothesised framework, multiple subthemes emerged from collected data for mobility but not for self-care.

#### Mobility

Sitting for too long was the frequently reported mobility problem by participants. They described that they cannot sit for a long time without feeling back pain and discomfort. This often led to a change in their position, either by standing or moving around. This was problematic in school, especially with lessons of a long duration without breaks, or where chairs had no back support. Further, standing for long time” and “walking for long distances” were also reported as the cause of back pain. Some participants avoided joining activities with friends and family that require walking a long distance.

*“Sitting down because I have two-hour lessons*, *instead of one hour*. *And sitting down for two hours is uncomfortable”…* “*Sometimes*, *I’m allowed a break like 10 minutes in between the lesson*. *But sometimes*, *I don’t*, *and it can get kind of painful”* (P6-Presurgery)

Bending activities (i.e., picking up things from floor or vacuuming) was described as painful and challenging activity. Those who had surgery or wore brace found that it was difficult to tie their shoelaces or put socks on. Avoidance behaviour, asking for help or finding alternative ways to perform the activity was reported.

*“When I keep having to bend down and pick things up*, *it’s quite hard to sometimes clean my room and especially vacuum…*.*and it hurts quite a lot at the end”* (P1-Observation)

“Bending over, that kind of thing; I tend to avoid bending over” (P2-post surgery).

Activities such as jumping and running at a pace that is more than a jog, were reported as difficult, and would be avoided.

*“The first time I realised that something was quite painful about it was when I was out with friends at a trampolining place*. *I started to jump*,.*this was the only time that it actually started to hurt*. *And I had to stop”* (P1-Observation)

Participants found that “carrying things” such as school bags could increase of back discomfort. Other reported that scoliosis “hindered their balance”, which make them feel dizzy and unable to walk in a straight line. Some adolescents reported that they were unable to go upstairs, and they have been allowed to use the lift instated of stairs at school. Side lying and difficulties finding a comfortable position have an impact on sleep quality as well.

*“Walking in straight*… *I can’t walk in like*… *well*, *I feel like I’m not walking in a straight line”* (P7- Pre-surgery)

*“Sometimes I do think it hinders my balance as well*, *so maybe that’s why I can’t stand up for very long periods of time without feeling dizzy*.*”* (P1- Observation).

#### Self-care

Dressing was the only challenging activity identified in this theme, causing discomfort for some participants. Activities such as washing oneself was discarded as no data supported its inclusion. Those who had undergone surgery reported needing help in self-care activities in early post-surgery period, but not after several months following surgery. Dressing also included tying shoes laces and putting socks on, which remained challenging following surgery.

*“Dressing*, *putting my socks on can be a bit of a task*, *or pulling up my trousers*, *but it’s not that hard that it’s a problem”* (P8-Post-surgery)

#### School-related activities

Participants reported that their focus and concentration during lesson time was impacted because of pain they felt with prolonged sitting. Thinking about pain and changing sitting position to find a comfortable spot affected concentration. Also, wearing a brace increased their discomfort and further affected their ability to focus.

*“Sometimes it’s definitely difficult to focus when my back is hurting that much and I’m trying to get on with the lesson*. *It is worse in science because we have these chairs*, *they don’t have a back on and so I have nothing to lean against*. *It makes me struggle because I’m trying to focus*, *but then trying to make sure that my back is not bending or hunching over because it would hurt more”*. (P1-Observation)

Participants reported that pain, surgery, and the follow-up appointments affected school attendance and performance. Surgery alone led up to 5 months away from school for one participant. For another participant she decided not to carry on schooling, because she had missed a lot of school days due to surgery.

*“It did affect my GCSEs because I literally had it done right before my exams*. *I had two months off school*, *…so I obviously missed a lot of school*..*now I still have follow-up appointments*, *obviously you miss time off school*.. *so it does add up”* (P5-Post-surgery)

### Psychological effects

Three main subthemes were identified related to this theme. Hypothesized factors such as self-esteem and positive feelings has been discarded, as no data supported its inclusion.

#### Emotional effects

Many terminologies were used interchangeably by participants with AIS to describe their negative feelings and emotions about scoliosis and it associated treatment (Fig 2). While no positive feelings were elicited. Participants used terms like “sad” when they felt pain, when they couldn’t explain their feelings to others, or when they think about surgery.

*“It makes me sad because it reminds me of being in pain a lot before the surgery*, *because the biggest thing I wanted out of the surgery was to help my pain*, *even if it was just a little bit”*. (P8-post-surgery)

Feelings nervous, worried and anxious were used when they compared themselves with others, or when thinking about the need of surgery and how it will affect their future life. They felt worried also because their parents felt worried too.

*“I feel a bit worried*, *like how it will affect me in the future and how it’s going to impact on my life*. *Just a bit nervous about if it will get worse in the future or not”* (P11-pre-surgery)

Participants reported feelings of being “annoyed” and, “irritated” because of having scoliosis or because they still have back problems following surgery. Wearing a brace made them feel annoyed as well. Feeling “afraid” was used when describing their emotions to have surgery, or that they might need surgery in the future. Disappointed was expressed when they believed there was nothing that could be done to treat their condition.

*“It can make me feel very annoyed*, *like it’s just a bit like why me*? *Why*?*”* (P10-presurgey)

“*I feel quite disappointed that there can’t be anything done*. *And I understand why there cannot be anything done*. *Yeah*, *I think just disappointment is the biggest one”* (P1- Observation)

#### Body image effects

This theme has a significant impact on participants lives and many subthemes were found. Participants reported feeling insecure about their body shape, making them unable to change when being with their friends or peers. They tend to hide their back from others by wearing large clothes. They felt that scoliosis limits wearing certain clothes such as a swimming kit, which limits participation in swimming. The term “dysmorphia” was used by one participant describing how she feels about her body shape.

*“I don’t know if this is the right thing*, *but it’s getting changed in front of people*, *I can’t do that and that’s the issue usually also in PE*, *… Or it’s when I’m with friends and we’re all getting ready for going out …*. *I just can’t get changed in front of them” (*P1- Observation).

This was also associated with preference of some of participants with AIS, to hide their condition from others.

“I didn’t tell anyone about it because I didn’t like it… I distanced myself from people.. I just wanted to be on my own. But it’s fine now” (P5-Postsurgey).

#### Mental effects

Participants described different methods to cope with pain, including taking analgesia, changing positions, and taking rest when pain was associated with activity.

*“It’s usually just a case of I just have to lie down for a minute*. *Obviously when I’m resting it doesn’t hurt*, *that’s literally the way to quickly cure it*, *is lie down” (*P5-Post-surgery).

Participants reported that they felt shocked and confused, because of the lack of knowledge about scoliosis and its treatment. They reported that they felt anxious and worried which affect quality of sleep.

*“I kind of had a stage of denial*, *I didn’t believe that it was real*, *and I felt that they were wrong*. *But then I saw the x-rays and I was shocked*, *to be honest*. *I didn’t realise what it actually looked like and what it was meant to look like basically”* (P1-Observation).

### Social effects

#### Participation

Sport and exercise participation was limited or discontinued due to back discomfort. Likewise, following surgery, some people were recommended to avoid contact sports, therefore their involvement in sports was limited.

*“I’ve always been a big sporty person*, *which is why it impacted me so much having scoliosis*. *I went from being very competitive and fast and loved sports to not being able to participate”* (P8-Posturgery).

Scoliosis also has an influence on "recreation and leisure" activities with family and friends. Going out with family and friends was difficult because of the severe pain.

“*Sometimes*. *When they were going trampolining*, *and I couldn’t’t go because it hurt that much*. *Or when it’s a birthday party and everybody is doing piggybacks and I have to say*, *hmm*, *maybe that’s not a good idea”* (P1-Observation).

#### Support

Friendship support was found to be important to participants and would assist in mental health, particularly during the recovery time following surgery.

*“My school has been amazing*.. *has provided a chair with lumbar support*. *It’s a very comfortable chair for me to sit in in exams*, *so I don’t have to sit for two hours to do a mock on an uncomfortable chair*.*”* (P8-Post-surgery).

"Mental health support" has been shown to be necessary since scoliosis has various effects on the psychological part of AIS life.

*“I think*, *definitely*, *the treatment you get post-op; you don’t get any mental support*, *and I think there should be more of it”* (P2-Post-surgery)

#### Satisfaction

Treatment satisfaction was essential to study participants. They felt that having observation without intervention was frustrating, and that performing exercises was not an effective treatment. Some were dissatisfied when surgery was the only possible option associated with long waiting periods.

“*So*, *I literally got diagnosed*, *they were like*, *“You’ve got this*,*” and then they were like*, *“We can’t help you with anything and you’ve just got to wait for surgery*.*” I’ve basically been given nothing*, *absolutely nothing”* (P10-Pre-surgery).

### Stratification analysis

Table 2 shows the stratification of data based on the participant’s management approach (i.e., observation, brace, pre-/ post-surgery). Although, some codes are not expressed by some participants it could be returned to individual variation in experience and personality. Overall, the majority of codes are expressed by participants across the three-management approach.

**Table 2:** Stratification of subthemes according to participants management approach.

Subthemes	Observation	Brace	Pre-surgery	Post-surgery
1a. Back hurt at rest	✓			✓
1b. Back hurt with brace		✓		
1c. Back hurt when exercising	✓	✓	✓	✓
1d. Back hurt because of surgery				✓
Hips hurt	✓	✓	✓	
leg ache/nerve pain			✓	
Shoulder aches	✓		✓	
Feel stiffened/inflexible	✓		✓	✓
Difficult breathing			✓	
Feel tired	✓		✓	
Uneven Shoulders	✓	✓	✓	✓
Uneven hips/waist	✓	✓	✓	
Leaning to one side			✓	
Ribs stick out			✓	
Time off school		✓	✓	✓
Focus during lessons	✓	✓	✓	
Ache with dressing	✓	✓	✓	✓
Jumping/jog	✓		✓	
Hinder balance/walking straight	✓		✓	
Bending	✓	✓	✓	✓
Carrying bags/things	✓		✓	
Going up stairs			✓	
Walking long distances			✓	
Sitting for long time			✓	✓
Side lying	✓	✓	✓	
Stand for too long	✓		✓	✓
Annoyed-irritated-frustrated	✓	✓	✓	✓
Sad	✓		✓	✓
Disappointed	✓			
Nervous-worry-anxious	✓		✓	✓
Afraid	✓		✓	
Feel insecure to change in front of others	✓		✓	
Hate body shape/dysmorphia			✓	
Limit wearing certain clothes			✓	
Hide back condition			✓	
Pain coping	✓	✓	✓	✓
Know about the scoliosis	✓	✓	✓	✓
Know about options of treatment	✓	✓	✓	✓
Sleep quality			✓	✓
School support		✓	✓	✓
Friends support	✓		✓	
Mental health support		✓	✓	✓
Participation In sport/physical activities/physical education	✓	✓	✓	✓
Participation in games /social activities with family friends	✓		✓	
Satisfaction about given treatment	✓	✓	✓	✓

### Comparison analysis

Table 3 presents the comparison and association performed between the codes elicited from the interviews and the items of the SRS-22r. Only one question from the SRS-22r was linked to the physical effects codes. Whereas other physical symptoms and body asymmetry were not linked to the any of the SSRS-22r items. Similarly, for activity effects only one question from the SRS-22r was linked to the codes, while three questions were linked to psychological effects cods and two questions to the body image codes. Participation was also linked to the SRS-22r and the satisfaction theme. Only 10 questions from the SRS-22r were connected to the codes raised from the qualitative data. This may indicate that the SRS-22r may not accurately reflect the perspectives of those with AIS.

**Table 3:** Comparison between SRS Scoliosis Research Society (SRS-22r) contents with themes, and subthemes elicited from interviews.

Qualitative data		SRS-22r
Themes	Subthemes	Questions
**Physical effects**	1a. Back hurt at rest	8- Do you experience back pain when at rest?
	1b. Back hurt with brace	
	1c. Back hurt when exercising	
	1d. Back hurt because of surgery	
Physical Symptoms	Hips hurt	
	leg ache/nerve pain	
	Shoulder aches	
	Feel stiffened/inflexible	
	Difficult breathing	
	Feel tired	
Body asymmetry	Uneven Shoulders	
	Uneven hips/waist	
	Leaning to one side	
	Rips stick out	
**Activity-related effects**	Time off school	17-In the last 3 months have you taken any days off work, including household work, or school because of back pain?
School-related activities	Focus during lessons	
Self-care	Ache with dressing	
Mobility	Jumping/jog	
	Hinder balance/walking straight	
	Bending	
	Carrying bags/things	
	Going up stairs	
	Walking long distances	
	Sitting for long time	
	Side lying	
	Stand for too long	
**Psychological-related effects**	Annoyed-irritated- frustrated	
	Sad	7-In the past 6 months have you felt so down in the dumps that nothing could cheer you up?
	Disappointed	16-In the past 6 months have you felt down hearted and blue?
Emotional effects	Nervous-worry-anxious	3-During the past 6 months have you been a very nervous person?
	Afraid from surgery/future with scoliosis	
Body image	Feel insecure to change in front of others	
	Hate body shape/dysmorphia	4- If you had to spend the rest of your life with your back shape as it is right now, how would you feel about it? 19. Do you feel attractive with your current back condition?
	Limit wearing certain clothes	
	Hide back condition	
Mental effects	Coping with pain	
	Know about scoliosis	
	Know about other treatment options	
	Sleep quality	
**Social effects**	Friends’ support	
Support	School support	
	Mental health support	
Participation	Sport/physical activities/physical education	
	Join games /social activities with family friends	18-Does your back condition limit your going out with friends/family?
Satisfaction	Satisfaction about given treatment	21-Are you satisfied with the results of your back management? 22-Would you have the same management again if you had the same condition?

## Discussion

This is the first qualitative study that has used in depth semi-structured interviews to evaluate the impact of scoliosis on the HRQOL of adolescents with AIS. The study was designed to evaluate the content validity of a currently used PROM (i.e., the SRS-22r) through a concept elicitation format, focusing on the adolescent experience with scoliosis and its associated treatment. Previous research studies have used quantitative methods to evaluate the validity of the SRS-22r [13, 14], with no attention being given to qualitative approaches. This study used words and phrases from participants with AIS in the generation of concepts, to ensure that the data are a true reflection of participants’ perspectives. When the qualitative data from participants with AIS was compared to the contents of the SRS-22r, a poor match was found indicating a lack of content validity of the SRS-22r. Furthermore, the terminology used in the SRS-22r does not reflect the language used by participants with AIS. Findings from this study provide strong evidence for the need to revise the SRS-22r, or development of a new PROM using data similar to that generated from this study that represents the voice of adolescents with AIS and is language and age relevant.

### Physical effects

Back hurt/pain was the most reported physical effect of scoliosis on HRQOL of participants, consistent with all previous reviews, where the prevalence of back pain amongst adolescents with AIS ranges between 37% to 42% [34]. Performing exercise improves HRQOL of AIS, by improving pain and function [35, 36]. However, most participants experienced back pain when exercising, which affected their adherence and participation in sport and PE at school. Consistent with previous studies, surgery contributed to back pain which may extend to two years post-surgery [37, 38]. This has been identified as the major concern in the presurgical period [22, 39]. The adolescents in this study used terms such as “hurt” or “ache” rather than “pain”, to describe their pain experience. This is not reflected in the terminology used by the SRS-22r [4], which may not be representative of this category of population. An important aspect of content validity is that the PROM should be age and language relevant [17, 40]. Other symptoms were also reported such as difficulty in breathing, stiffness, hip, and shoulder pain in accordance with earlier reports [22, 41, 42]. However, these symptoms are not assessed by the SRS-22r. Body asymmetry was also reported by participants as a major concern consistent with previous studies, where body asymmetry associated with scoliosis had a social and psychological impact on life [43]. This aspect is not assessed by the SRS-22r. These findings reflect the strength of this study in using a concept elicitation interview to capture participant’s own perceptions of their condition to inform content validity [24].

### Activity-related effects

Carrying out normal daily activities such as sitting, standing and walking, if performed for a long time, was reported to aggravate symptoms. These activities were reported in a previous qualitative study in a presurgical period [22]. Scoliosis also affects standing stability and balance [44]. Walking in a straight line was altered because of scoliosis, which has been assessed in earlier studies [45]. Bending was limited generally, and for those who had a surgery in particular, since spinal mobility and flexibility is reduced following surgery [46, 47]. Participants reported discomfort when carrying backpacks, which has been explained by the increase in the compressive forces on the curve apex [48], that may cause additional strains on muscles around the spine. Jumping was avoided as they realised that it caused back pain [22]. Going up stairs was also reported as difficult, although it is not reflected in any scoliosis literature. The association between performing functional tasks such as climbing stairs and having back pain has been previously noted in other groups [49]. Participants described that their focus during lesson time was affected because of a feeling of back hurt during sitting for prolonged time. A study on the association between pain and functioning in school found that adolescents with pain were more likely to have low school engagement, attendance and performance [50]. Participants also reported that they missed school days because of feelings of pain, surgery or hospital appointments which affected their academic achievements. In some countries, surgery is performed during summer time to reduce distribution on school performance [51]. However, for some people with AIS, time off school may extend to 6 months [12, 52]. Regarding self-care, participants reported that dressing caused aching and was a challenging. In summary, these findings demonstrated the significance of these aspects to HRQOL of adolescent with AIS, and therefore should be reflected in a PROM that evaluates HRQOL of this population.

### Psychological effects

The emerged themes revealed that scoliosis has a significant impact on the psychological aspect of individuals with AIS life. Different negative feelings were reported associated with both diagnosis and treatment. Feelings of frustration, irritation, and annoyance as well sadness, when experiencing pain were described by participants [12]. Disappointment was also discussed, relating to the treatment decisions, as mentioned in a recent review [18]. Worry and fear about the need for surgery and a future living with scoliosis contributed to feelings of nervousness and anxiety, as discussed before [18, 22, 53], and is also reported by the parents of adolescents with AIS in other studies [19, 21, 39]. Different questions in the SRS-22r evaluated mental health of adolescents, however, they are driven from the SF-36 which is an adult rather than a paediatric questionnaire [1].

The effects of scoliosis on body image of adolescents with AIS is another important factor found is this study. Participants disclosed feeling insecure to change in front of others and hid their back. Scoliosis also limited their ability to wear certain clothes [18, 22]. Body dysmorphia and hate of body shape were raised by one participant with a severe curve. This was associated with dissatisfaction about body image [54]. The self-image domain in the SRS-22r assesses the effect of AIS on body image [4]. Only two questions from this domain seems relevant to the driven subthemes.

A pain coping subtheme was revealed by our participants. A prior study found that coping with pain is dependent on the personality of the individual and affects the recovery process [51]. Since the HRQOL of adolescents with AIS may be related more to psychosocial effects than to physical effects and its consequences [55], it is recommended to train clinicians who are caring of individuals with AIS, in stress reducing techniques including pain coping strategies [51, 56]. Information about scoliosis, and the options for treatment and associated consequences, has a psychological impact on participants life. This has been studied among adolescents with AIS and their parents prior to surgery [22, 39], wherein providing support and information to adolescents and their families helps in reducing stress and anxiety associated with scoliosis [18, 51].

Participants also reported that their sleep quality was affected by scoliosis. A recent study assessed the sleep profile of adolescents with AIS and revealed poor sleep quality associated with high pain intensity [57]. Furthermore, following surgery reports indicated anxiety, nightmares and sleeping difficulties, both after the hospital visit and for a long time after the recovery period [20]. These results highlight the significant impact of scoliosis on the psychological aspects of life with AIS and therefore, should be included in a PROM evaluate their HRQOL.

### Social effects

Participation in activities of daily life is important for the physical and psychological development of adolescents [58]. In this study, participants reported reduced participation in social and sport activities consistent with previous studies [59]. Fear of pain or injury, and reduced self-image, may limit social participation, and has a negative impact on wellbeing [20]. Spinal fusion surgery has been shown to have a long term effect on an individuals’ social life, and that providing support in the form of information and coping techniques increased the level of social participation [51]. In our study, participants reported the need for mental health support to overcome the psychological consequences of scoliosis and surgery. Earlier studies recommended providing psychological support to adolescents with AIS and their parents to minimize stress and uncertainty about surgery [20, 21]. Satisfaction about the treatment, assessed based on the answers of the SRS-22r was relevant to our study participants. Two questions in the SRS-22r assess satisfaction and were linked to the identified themes.

### Strengths and limitations

This study is reported in line with published guidance of qualitative research (COREQ) [23], and is based on a published protocol [1] to ensure rigor and comprehensiveness of the findings. Findings of this study using concept elicitation format allow capturing participant’s own perceptions of their condition to inform content validity [24]. Furthermore, patient perspective was central in this study, through involvement of PPI representative (ER) in the study design and interpretation of the results.

Limitations are in a number of areas with several factors that have influenced data collection and analysis, and thus may influence the findings reported. The sample was recruited from a single hospital in the UK limiting transferability of the findings. However, we assured diversity in population characteristics using a heterogenous purposive sampling technique [29], which ensures the recruitment of participants with various demographic, clinical characteristics, and treatment approaches. The children/adolescents were not talkative, tended to give short answers and were reluctant despite prompting to elaborate on their responses and give further details. The assessment of concept saturation was performed following data collection, although, it has been recommended to be evaluated during data collection [28]. Coding of data was performed by one researcher (SA) and evaluated later by the research team. However, the coding framework and the codebook provide an evidence that coding is a true reflection of participants quotes [24].

### Implications for practice and future research

The findings highlight that the content of the SRS-22r does not adequately capture the experience of adolescents with AIS and the effect of scoliosis on adolescent HRQOL. Themes and codes driven from participant data should inform the adaptation of the SRS-22r, or the development of a new PROM, which is relevant to AIS and based directly on qualitative input from adolescents. Furthermore, qualitative research is needed to assess the content validity of the developed PROM through conducting cognitive testing to test its relevance, comprehension, and comprehensiveness from both adolescent and practitioner perspectives [6].

## Conclusion

Concept elicitation interviews with adolescents with AIS revealed that scoliosis and its associated treatment has a broad impact on life, including physical, activity-related, psychological, and social effects. Evaluation of content validity of SRS-22r by comparing between the concepts and codes elicited from interview data, and the SRS-22r contents, revealed that the SRS-22r does not accurately reflect the words and phrases of adolescents with AIS, indicating a lack of its content validity. Themes and subthemes that have resulted from this qualitative study could be used to develop a new PROM for AIS or update the existing SRS-22r to enhance its use for this population.

## Supporting information

S1 Appendix Topic guide. (PDF)

S2 Appendix Saturation table. (PDF)

S3 Appendix Themes, subthemes, codes. (PDF)
