# Supplementary material for: Content validity of the Scoliosis Research Society questionnaire (SRS-22r): A qualitative concept elicitation study
Source: PLoS One. 2023 May 5;18(5):e0285538. doi: 10.1371/journal.pone.0285538 (PMC10162511; doi:10.1371/journal.pone.0285538)
Supplement: S1 Appendix — (PDF) [file pone.0285538.s001.Pdf]

Topic guide pilot tested in advance of the main data collection

Ethics Statement/ instructions

Firstly, I would like to thank you for agreeing to take part in this interview. “My name is Samia. I am a student in University of Birmingham. I am also a mother and one of my kids is the same age as you. Today, I will be chatting with you about your back.

Just a reminder that this interview will be audio/video-recorded, but all information will be kept confidential and anonymised, so you are not identifiable from the questions/answers you give during the recording. If you change your mind at any point, just tell me that you want to stop the interview; it is absolutely fine to do that. This will not affect your current and future health care.

You also have the right not to answer a question if you do not wish to. There are no right or wrong answers. I am interested in your own experiences and thoughts. If I ask you a question and you don’t know what I mean, please just say ‘I don’t get it’ or ‘I don’t know what you mean,’ and I’ll try to ask it another way.

Just to reassure you- none of the answers or information that you give me will be associated with you and your name will be replaced by code number when we report the findings. Once the information from the interview has been used, the recordings will be deleted. Before we start, is there anything you’d like to ask?

Background

- As you have seen in the information sheet, the SRS-22r is a questionnaire that assesses quality of life in people with scoliosis. To remind you quality of life is measure of health, comfort, and ability to participate or enjoy life events. We would like to hear how you feel about your back, and how you feel it affects your life. Can I confirm that you have read and understand the information sheet and signed the consent/assent form? Do you have any questions?

Introductory Questions

- Can you tell me a bit about yourself? (How old are you ? in which year at school you are? Do you have any brothers and sisters? What is your favourite subject?)
- What do you like to do for fun or in your spare time? (Do you like to go for a walk, or other activities like exercise?)
- Which type of exercise is your favourite? (Basketball, ballet, swimming? What do you like/dislike about exercising?)

Concept elicitation

- Thinking about your back, do you or have you ever had any difficulties ? (Are there any further issues that you can think of? (For example, your body is hurting you; pain at your joints; You feel pain during or after doing exercise/PE; chest pain; difficult breathing, decrease in your movement range? How is that?)

- How many times do you take pain medications? (Is it effective? What do you do to ease pain other than taking medication?)
- If we want to measure the pain that you usually have. How much would you give it, in a scale from 0 to 10 ?
- Is there any specific part of your body you feel/think is affected because of your back? What is this part? how it is has been affected by your back?
- Can you tell a bit more about any difficulties you have with your back? Which aspects of your daily life does it affects? (Walking to school/catching the bus, climbing stairs, carrying bag, sitting, standing, bending, kneeling)
- Can you explain for me if and how your back affects you at school and activities linked to school? (Studying/doing your homework, working in lab/art/music class)
- Do you participate in PE/school sports? How do you feel about doing organised exercise or sports with your classmates?
- Have you ever had off days due to your back? Why?
- How your back affect your ability to do activities to go out with family or friends? (Visiting friends?)
- How your back affect your ability to do things around home? (Playing sport or joining a game ?)
- How your back affect your ability to do things at home ? (How your back affect you on taking care of your body parts, eating, bathing, toileting, dressing? Tidy up your room, helping family in preparing meals, assisting others, taking care of pets/plants).
- Can you describe your feelings when you have an issue with your back, how does this make you feel? Does it worry you at all? Do you know what it is specifically that worries you? (Fear of pain, difficulty preforming your homework, your scores in school less than usual, limited in activities that you can do). Does this affect your energy? Does your back condition ever make you feel down or discouraged, afraid, angry, or anxious ? Does is it affect your ability to sleep?)
- How do you feel about the shape of your back? how do you think it is affecting your life? how it is limiting your ability to do things? How is it affect your relationship with other?
- Can you tell me what management/treatment you have received for your back? What was the treatment How do you think/feel about it ? Do you have an idea about other options of treatments you might receive for your back ? what is it ? How do you feel if you have the same treatment again?
- Can you describe the support that you have received during your treatment? Which type of support have you received ? from whom? (Family, friends, peers, health professionals (doctors, nurses, physiotherapists)? What do you think about it?
- What do you feel would improve your experience of living with scoliosis? what is it?
- Is there anything related to your experience with scoliosis we have not talk about it and would like to bring it up now?
