# Supplementary material for: Content validity of the Scoliosis Research Society questionnaire (SRS-22r): A qualitative concept elicitation study
Source: PLoS One. 2023 May 5;18(5):e0285538. doi: 10.1371/journal.pone.0285538 (PMC10162511; doi:10.1371/journal.pone.0285538)
Supplement: S2 Appendix — (PDF) [file pone.0285538.s002.pdf]

S2. Saturation table showing that concept saturation was achieved

Themes & subthemes	Subtheme	Interview number										
		1	2	3	4	5	6	7	8	9	10	11
**Physical effects**												
**Physical Symptoms**	**1a. Back hurt at rest**		✓									
	**1b. Back hurt with brace**			✓								
	**1c. Back hurt when exercising**	✓										
	**1d. Back hurt because of surgery**		✓									
	**Hips hurt**						✓					
	**Nerve pain, leg ache/weak**							✓				
	**Shoulder aches**	✓										
	**Feel stiffened/ inflexibility**	✓										

	**Feel tired**	✓										
	**Difficult breathing**							✓				
**Body asymmetry**	**Uneven Shoulders**	✓										
	**Uneven hips/waist**		✓									
	**Leaning to one side**		✓									
	**Rips stick out**							✓				
**Activity-related effects**												
**School-related activities**	**Time off school**		✓									
	**Focus during lessons**	✓										
**Mobility**	Jumping/jog	✓										
	Hinder balance/walking straight	✓										
	Bending	✓										

	Carrying bags/things	✓										
	Going up stairs							✓				
	Walking long distances		✓									
	Sitting for long time	✓										
	Side-lying	✓										
	Stand for too long	✓										
**Self-care**	Ache with dressing	✓										
**Psychological related effects**												
**Emotional effects**	Annoyed-irritated- frustrated	✓										
	Sad	✓										
	Disappointed	✓										
	Nervous-worry-anxious	✓										

	Afraid				✓							
**Body-image**	Feel insecure to change in front of others	✓										
	Hate body shape							✓				
	Limit wearing certain clothes					✓						
	Hide back condition					✓						
**Mental effects**	Pain coping strategies	✓										
	Sleep quality		✓									
	Know about scoliosis	✓										
	Know about options of treatment	✓										
**Social effects**												
**Support**	From school			✓								
	From friends		✓									

	Mental health support		✓									
**Participation**	In sport/physical activities/physical education	✓										
	Join games/social activities with family/friends	✓										
**Satisfaction**	Satisfaction about given treatment (surgery, physiotherapy, brace)	✓										
**No. of new codes appearing in each interview**		**25**	**9**	**2**	**1**	**2**	**1**	**5**	**0**	**0**	**0**	**0**
**% Of total new codes (Total =45)**		**55.5**	**20**	**4.4**	**2.2**	**4.4**	**2.2**	**11.1**	**0**	**0**	**0**	**0**
