# Supplementary material for: Content validity of the Scoliosis Research Society questionnaire (SRS-22r): A qualitative concept elicitation study
Source: PLoS One. 2023 May 5;18(5):e0285538. doi: 10.1371/journal.pone.0285538 (PMC10162511; doi:10.1371/journal.pone.0285538)
Supplement: S3 Appendix — (PDF) [file pone.0285538.s003.pdf]

S3. Theme, subthemes, codes		
**Theme**	**Subtheme**	**Codes “Participants quotes”**
**Physical effects**		
**Physical Symptoms**	1a. Back hurt at rest	‘It really depends, to be honest. There’s nothing in particular which brings it on. It just happens. It can just happen. I might be just at rest, sat in a chair for too long, and it can hurt’ (P2)
	1b. Back hurt with brace	‘The only pain I get is because of my brace’ (P3)
	1c. Back hurt when exercising	‘Sometimes it hurts my back when I do some exercises …. Like at times, when I play football, it hurts my back and if I do too many exercises and stuff, it starts to make my back hurt.’ (P11)
	1d. Back hurt because of surgery	‘I wouldn’t have been able to exercise or walk to help the pain before surgery, because that was what was causing my pain, whereas now if I do get pain, it’s because of the surgery’ (P8)
	Hips hurt	‘My hips, I guess, my hips start hurting a bit but not as noticeable as my back’ (P6)
	leg ache/nerve pain	‘I leg-ache a lot, but we don’t what that is caused by’ (P8)
	Shoulder aches	‘I think just my shoulders and my hips sometimes because it’s kind of like disjointed everything. They ache most of the time and no matter how many medications I can take for it, it’ll always be there because it just reoccurs’ (P1) ‘I think, when I wake up in the morning, they’ll be really tight, and they’ll be aching.’ (P6)
	Feel stiffened/inflexible	‘I’m less flexible; I’ve never really been that flexible. I used to be really; I could do anything. But now it feels kind of stiffened and I’ve definitely lost quite a lot of flexibility and sometimes mobility’ (P1)
	Difficult breathing	‘It makes my lungs hurt from running, so then it makes it difficult breathing.’ (P7)
	Feel tired	‘I feel more tired than other people after I do a lot of things and I have to take more breaks than most of my friend’s when they do the same things. So usually, I’m just way more tired.’ (P1)

**Body asymmetry**	Uneven Shoulders	“She’d noticed that my shoulders were uneven, and she tried to like straighten them but they wouldn’t straighten” (P10)
	Uneven hips/waist	‘I was 13, but for a year I had noticed that one side of my waist was straight and the other side was going in like that.’ (P8)
	Leaning to one side	“I was constantly in a state of leaning to the one side. It was quite apparent as soon as I was diagnosed” (P2)
	Rips stick out	“that’s when my back started getting worse and I noticed my rib would stick out and it progressively got a lot worse, pain and looks-wise, and I just wasn’t happy with my back at all. I was very upset” (P8)
**Activity related effects**		
**School-related activities**	Time off school	‘I had a lot of time off. I was off for five months.’ ‘Before the surgery I was off every day pretty much. I would go to school for an hour or two and the pain would get so bad that I’d have to call Mum or Dad to come and pick me up.’ (P8)
	Focus during lessons	‘Sometimes it’s definitely difficult to focus when my back is hurting that much and I’m trying to get on with the lesson. It’s worse in science because we have these chairs, they don’t have a back on and so I have nothing to lean against. It makes me struggle because I’m trying to focus, but then trying to make sure that my back isn’t bending or hunching over because it would hurt more.’ (P1)
**Self-care**	Ache with dressing	‘Sometimes dressing, sometimes it’s – not painful, but really aching and I just can’t be bothered. It’s just quite a challenge every morning.’ (P1)
**Mobility**	Jumping/jog	‘Running. Football, I’ve played a bit of football since, it’s a bit difficult. Anything kind of above a jog, it’ll sometimes start hurting.’ (P6) ‘I started to jump, I’d been many times before, but this was the only time that it actually started to hurt.’ (P1)
	Hinder balance/walking straight	‘I do think it hinders my balance as well, so maybe that’s why I can’t stand up for very long periods of time without feeling dizzy.’ ‘Walking in straight... I can't walk in like... well, I feel like I'm not walking in a straight line’ (P7)

	Bending	‘Bending over, that kind of thing; I tend to avoid bending over. If I have to tie my shoes or something like that, I’ll put my leg up on a chair. I don’t tend to bend down as such’ (P2) ‘It’s because I can’t bend my back, like how you would bend to stretch your toes, I can’t bend like that with the brace on ‘ (P3)
	Carrying bags/things	‘I had like a handbag for school, so obviously there’s more pressure on one side and I used to have to get my friends to carry them because my back wasn’t strong enough’ (P5) ‘I think sometimes if I had to, let’s say, carry a heavy basket of washing or something, I’d potentially struggle with that’ (P2)
	Going up stairs	‘Pain mostly. Walking in straight... I can't walk in like... well, I feel like I'm not walking in a straight line. But then... And then walking far distances and going up stairs.’ (P7)
	Walking long distances	‘And then walking far distances.’ (P7) ‘I guess walking for like a while, it’ll be noticeable, but short distance is not too bad’ (P6).
	Sitting for long time	‘When doing sports activities, it causes pain in my back and sitting down for long periods of time also affects it’ (P11).
	Side lying	‘At the start it used to be really bad, every single time I lay in a certain position, and it started to really ache.’ (P1)
	Stand for too long	‘If I have to stand around for too long, it starts to really hurt and then I have to immediately sit down.’ (P1) ‘Before, not really. But since, it hurts to sit down or stand up for too long, I guess. (P6)
**Psychological effects**		
**Emotional effects**	Annoyed-irritated-frustrated	“It makes me feel annoyed because most people don't have this, but what can you do?’ (P3) ‘I'm quite moody. Sometimes I'm stroppy about it and I'm annoyed…oh irritated” (P7)
	Bad/sad/bothering	“It makes me sad because it reminds me of being in pain a lot before the surgery, because the biggest I wanted out of the surgery was to help my pain, even if it was just a little bit” (P8)
	Disappointed	“I feel quite disappointed that there can’t be anything done. …. Yeah, I think just disappointment is the biggest one” (P1)
	Nervous-worry-anxious	‘I’m nervous about having the surgery’. ‘I feel a bit worried, like how it will affect me in the future and how it’s going to impact on my life. Just a bit nervous about if it will get worse in the future or not’ (P11).

	Afraid from surgery/future with scoliosis	‘I didn’t really want it to get bad like my sister’ (P4)
**Body image**	Feel insecure to change in front of others	‘I wouldn’t get changed in front of others. I’d go in the toilet cubicle when we were getting ready for PE. I wouldn’t get dressed with all the other girls. I’d go somewhere separately. I’d have to wear a jacket with everything, or a jumper, and even if I was covered up, I was still very insecure.’ (P8)
	Hate body shape	‘I hate it. It’s given me the worst body dysmorphia’ (P10)
	Limit wearing certain clothes	‘I’d rather not swim, I guess. I don’t hate swimming but just wearing shorts and my back is like… I’d rather not show it.’ (P6)
**Mental effects**	Pain coping	‘It’s usually just a case of I just have to lie down for a minute. ..that’s literally the way to quickly cure it, is lie down.’ (P5) ‘If I feel the pain coming on, I’ve got a routine I get on to, “Okay, this is coming on; before it gets worse, let me just rest for 10 minutes and then see how I feel.’ (P2)
	Know about scoliosis	‘I think it was just the fact that, in the early, early days, I just did not have a clue how that was going to affect my life.’ (P2)
	Know about options of treatment	“I remember the first time I went to the appointment, the person basically said, you’re eligible for surgery now. And I was like, what? Obviously, we didn’t even know what it was” (P5-Post-surgery).
	Sleep	‘I think my sleep affects me mentally, from anxiety more than it does pain. It’s more the mental side effects from the scoliosis than the physical.’ (P2)
**Social effects**		
**Support**	From school	‘So, I have a time out card at school, meaning I can have a walk outside the classroom for like five minutes. And then I also have a five-minute early pass, so I leave five minutes early from lessons, so the other lesson.’ (P7)
	From friends	‘I look back on it, I would have definitely told my friends. Because when I did tell them eventually, literally when I got told that I was having it and they were all fine and supportive and things like that. When I look back on it, I think why you not just told them’ (P5)

	Mental health support	‘Probably would have been good for them to think about the mental effects as well, because obviously, spines and surgery, that kind of thing, they are just focused on the metalwork and whether the actual back is okay. But I don’t feel like anybody really bothered about how I was feeling…And I think, definitely, the treatment you get post-op; you don’t get any mental support.’ (P2)
**Participation**	In sport/physical activities/physical education	‘It definitely stops a lot of things to do with PE as well because there’s a lot of physical activities, mainly like yoga and things, because they always say bending in all these different directions. But I’m not that flexible and I can’t do that.’ (P1) ‘I guess I can’t really do like sports. The amount of sports I’m able to do comfortably has kind of narrowed.’ (P6)
	Join games /social activities with family friends	‘Sometimes it affects it, like say you want to go on like walks or something for a long period of time, then it would hurt’ (P11) ‘I couldn’t go out with my friends or anything, if they asked me to go out or sleep over. It just restricted me to stay at home because I physically couldn’t do anything I was in that much pain and I was very, very deformed’. (P8)
**Satisfaction**	Satisfaction about given treatment	‘I would comply with what they say, but I would feel quite disappointed because I would have to do it all over again and have to go through the same process and feel quite uncomfortable while doing it.’ (P1) ‘They obviously don’t give you a brace because they’re like, “Your curve is so big it’s not going to work.” They basically give up on you and say, “You’ve got to wait for surgery,” (P10) ‘Much better about my back shape, because it was getting to a point before my operation, and the curve was getting worse and worse, and I was getting shorter and shorter. So now I feel a lot better. I feel a lot more upright, like I stand a lot taller.’ (P2)
